# Preparation of A Spaceflight: Apoptosis Search in Sutured Wound Healing Models

**DOI:** 10.3390/ijms18122604

**Published:** 2017-12-03

**Authors:** Stefan Riwaldt, Monica Monici, Asbjørn Graver Petersen, Uffe Birk Jensen, Katja Evert, Desiré Pantalone, Kirsten Utpatel, Matthias Evert, Markus Wehland, Marcus Krüger, Sascha Kopp, Sofie Frandsen, Thomas Corydon, Jayashree Sahana, Johann Bauer, Ronald Lützenberg, Manfred Infanger, Daniela Grimm

**Affiliations:** 1 Department of Biomedicine, Aarhus University, 8000 Aarhus C, Denmark; sr@biomed.au.dk (S.R.); aspe@biomed.au.dk (A.G.P.); stf@biomed.au.dk (S.F.); corydon@biomed.au.dk (T.C.); jaysaha@biomed.au.dk (J.S.); 2 University Clinic for Plastic, Aesthetic and Hand Surgery, Otto-von-Guericke-University, 39120 Magdeburg, Germany; markus.wehland@med.ovgu.de (M.W.); marcus.krueger@med.ovgu.de (M.K.); sascha.kopp@med.ovgu.de (S.K.); ronald.luetzenberg@med.ovgu.de (R.L.); manfred.infanger@med.ovgu.de (M.I.); 3 ASA Campus Joint Laboratory, ASA Research Division, Department. of Experimental and Clinical Biomedical Sciences, University of Florence, 50121 Florence, Italy; monica.monici@unifi.it; 4 Department of Clinical Genetics, Aarhus University Hospital, 8000 Aarhus C, Denmark; uffejens@rm.dk; 5 Department of Clinical Medicine, Aarhus University, 8000 Aarhus C, Denmark; 6 Institute for Pathology, University of Regensburg, 95053 Regensburg, Germany; katja.evert@klinik.uni-regensburg.de (K.E.); kirsten.utpatel@klinik.uni-regensburg.de (K.U.); matthias.evert@klinik.uni-regensburg.de (M.E.); 7 Department of Critical Medicine and Surgery, University of Florence, 50134 Florence, Italy; desire.pantalone@unifi.it; 8 Department of Ophthalmology, Aarhus University Hospital, 8000 Aarhus C, Denmark; 9 Max-Planck-Institute for Biochemistry Martinsried, 82152 Planegg, Germany; jbauer@biochem.mpg.de

**Keywords:** skin, wound healing, suture, apoptosis, caspases, extracellular matrix proteins

## Abstract

To prepare the ESA (European Space Agency) spaceflight project “Wound healing and Sutures in Unloading Conditions”, we studied mechanisms of apoptosis in wound healing models based on ex vivo skin tissue cultures, kept for 10 days alive in serum-free DMEM/F12 medium supplemented with bovine serum albumin, hydrocortisone, insulin, ascorbic acid and antibiotics at 32 °C. The overall goal is to test: (i) the viability of tissue specimens; (ii) the gene expression of activators and inhibitors of apoptosis and extracellular matrix components in wound and suture models; and (iii) to design analytical protocols for future tissue specimens after post-spaceflight download. Hematoxylin-Eosin and Elastica-van-Gieson staining showed a normal skin histology with no signs of necrosis in controls and showed a normal wound suture. TdT-mediated dUTP-biotin nick end labeling for detecting DNA fragmentation revealed no significant apoptosis. No activation of caspase-3 protein was detectable. *FASL*, *FADD*, *CASP3*, *CASP8*, *CASP10*, *BAX*, *BCL2*, *CYC1*, *APAF1*, *LAMA3* and *SPP1* mRNAs were not altered in epidermis and dermis samples with and without a wound compared to 0 day samples (specimens investigated directly post-surgery). *BIRC5*, *CASP9*, and *FN1* mRNAs were downregulated in epidermis/dermis samples with and/or without a wound compared to 0 day samples. *BIRC2*, *BIRC3* were upregulated in 10 day wound samples compared to 0 day samples in epidermis/dermis. *RELA/FAS* mRNAs were elevated in 10 day wound and no wound samples compared to 0 day samples in dermis. In conclusion, we demonstrate that it is possible to maintain live skin tissue cultures for 10 days. The viability analysis showed no significant signs of cell death in wound and suture models. The gene expression analysis demonstrated the interplay of activators and inhibitors of apoptosis and extracellular matrix components, thereby describing important features in ex vivo sutured wound healing models. Collectively, the performed methods defining analytical protocols proved to be applicable for post-flight analyzes of tissue specimens after sample return.

## 1. Introduction

In the near future, more space exploration programs and commercial spaceflights will lead to longer space missions, an elevated number of crew members and space travelers on board, as well as an increase in on-board and extravehicular activities. This will elevate the risks of traumatic injuries and emergencies in space. A critical aspect in surviving a trauma or a surgery is sufficient wound healing, which depends on a healing process and suture behavior in space.

Wound healing is a complex process consisting of three partially overlapping phases: inflammation, tissue formation, and tissue remodeling [1,2]. Hence, it involves a series of cell populations that play specific roles at different stages of the repair process, e.g., immune cells are crucial in the early inflammatory phase, when their proliferation strongly increases [3,4].

A proper sequence and timing of events is very important for a successful repair. Among various biochemical and physiological factors affecting wound healing, the mechanical factors play a significant role. These are skin tension, mechanical forces at the wound margins and mechanical stress produced by stitches (when a suture is necessary). In addition, wound contraction due to myofibroblasts has a strong influence on the development of the repair process and scar quality [5]. During the different phases of wound healing, apoptosis balances cell growth and acts to eliminate cell populations that already completed their specific tasks without causing tissue damage or inflammatory reaction [6,7].

As mentioned by Drudi et al. in their “state of the art” report concerning space surgery from 2012 [8], tissue repair mechanisms, wound healing and suture behavior have been poorly studied in the space environment, characterized by unloading conditions. The literature on these topics, while certainly not exhaustive, reports conflicting results [9,10]. However, the various studies indicate that weightlessness can induce impairments in repair processes [11,12,13,14] and alters the behavior of the cell populations involved in tissue repair [15,16,17]. The risk of wound healing complications following traumatic events and emergency surgery in space is therefore an important health concern for space agencies.

The project “Wound Healing and Sutures in Unloading Conditions”, selected by the European Space Agency (ESA) in the context of the International Research Announcement for Research in Space Life Sciences (ILSRA-2014) at the International Space Station (ISS), aims to study the behavior and healing of wounds and sutures in unloading conditions in space. The project is based on human tissue cultures from consenting patients who underwent abdominoplasty. The small tissue specimens are secured to suitable frames to apply a tension similar to the physiological conditions and subsequently wounded and sutured.

The study here presented is part of activities aiming to prepare the inflight space experiment. The principal aim is the evaluation of apoptosis and necrosis in skin tissue samples cultured in serum-free medium for 10 days.

Human skin cultures had been extensively studied because skin autografts are used to treat severe burns. Therefore, preservation conditions and tissue viability are very important for optimal clinical results. Even if the storage progressively affects the cellular component of the tissue, suitable temperature values and culture media allow to maintain an acceptable tissue viability for more than 4 weeks, while a significant deterioration had been observed after 35 days [18].

Full-thickness skin cultures can be also regarded as interesting scientific model for wound healing studies. To carry out experiments on the ISS successfully, it is very important to ascertain the survival of the samples for a sufficient amount of time, i.e., at least 10 days. The assessment of apoptosis and necrosis is considered a valuable way to obtain information on tissue damage and its adaptation to the in vitro conditions [19,20]. Moreover, since apoptosis is involved in the regulation of wound healing [6], the monitoring of apoptosis in wound healing models can provide insights into the evolution of the repair process.

The first phases of wound repair comprise haemostasis (platelet accumulation, coagulation) and inflammation, which is characterized by migration of immune cells and fibroblasts in the wounded area. A fibrin–fibronectin clot is detectable in areas of active bleeding after injury [21]. It interacts with various cells involved in wound repair and plays a key role in extracellular matrix (ECM) formation. Great amounts of cytokines and growth factors are produced, fibroblasts and keratinocytes take an activated phenotype [2]. Neutrophils entering the wound area eliminate microorganisms, then they will become apoptotic, and will be finally consumed by macrophages, a process without further inflammation. Macrophages, play a fundamental role in the transition between inflammation and repair [22]. In this early phase, the process of apoptosis will start as early as 12 hours after wound injury [23].

The following intermediate stage (proliferation phase) is characterized by keratinocyte proliferation and migration. In addition, fibroblasts proliferate, ECM is synthesized and deposits are visible, and the process of angiogenesis is started. Apoptosis is important for normal wound healing, especially in the removal of inflammatory cells and others like endothelial cells and the transformation of granulation tissue into scar tissue [22]. These cell populations are rapidly proliferating during tissue repair. The process of apoptosis keeps cell growth in balance. Apoptosis in myofibroblasts is initiated on day 12, peaks at day 20, and is resolved at day 60 [23]. Normal mice exhibit an inverse relationship between *BCL2* and *p53* over time. Directly after injury, the *BCL2* mRNA is elevated and *p53* down-regulated to allow proliferation of the cells, which is an important step for wound repair [24]. During the time course, *BCL2* levels were down-regulated, while contrarily the *p53* mRNAs were up-regulated to reduce the proliferative response.

The late stage of wound healing (tissue remodelling) is characterized by ECM remodelling with scarring. The complete biological process is tightly controlled by multiple cell types secreting growth factors, cytokines, and soluble factors into the interstitial space to achieve a closure and a functional restoration of the barrier. The apoptosis of the involved cells is a key factor for wound repair regulation.

Due to ex vivo culturing conditions, serum- and blood-derived signaling molecules and immune cells are absent in this approach. Thereby, a direct deduction of our findings on known functions of apoptosis in wound healing is not applicable [6]. As already applied in further studies [25], our primary focus is apoptosis, as a surrogate for cell viability. Nevertheless, stated findings display new insights in cell viability and the interplay of apoptotic effector proteins on ECM proteins and NF-κB. An essential role of fibronectin is indicated for all phases of wound healing [21]. Thus, fibronectin is known to affect inflammation by modulating macrophage activation [1]. Whereas, an increase in fibronectin gene is found in abnormal wound healing, e.g., keloids [26]. Fibronectin influences ECM-functionality, by its connection to further essential ECM proteins, e.g., actin or laminin, via integrin receptors [27].

To prepare the future ESA spaceflight mission, we searched for apoptosis and necrosis and studied mechanisms of apoptosis in wound healing models based on ex vivo skin tissue cultures. Therefore, this study aims (i) to characterize the morphological changes and to test the viability of the tissue specimens; (ii) to determine the gene expression of activators and inhibitors of apoptosis with focus on the extrinsic and intrinsic pathways of apoptosis and extracellular matrix (ECM) components in wound and suture models, including to analyze the interaction network of the genes of interest using STRING (Search Tool for the Retrieval of Interacting Genes/Proteins) analysis and (iii) to design the analytical protocols which will be performed in the future on tissue specimens after the post-spaceflight download of the samples.

## 2. Results and Discussion

Providing adequate medical care in space, close to the terrestrial standards, is one of the challenges to be addressed in future space exploration programs. Emergency and space surgery is a relatively little studied area of space medicine that will be increasingly important for the future of space exploration with long-term missions. The purpose of the project “Wound Healing and Sutures in Unloading Conditions” is to get insights, which contribute to fill this gap. However, the in-flight experiment—that is the monitoring of suture behavior and healing in unloading conditions—requires meticulous preparation and preliminary studies on ground. An important prerequisite for the success of the experiment is the viability of the suture models for a sufficiently long time.

### 2.1. Characterization of the Morphological Changes and Testing the Viability of the Tissue Specimens

To answer the question, whether skin samples obtained from patients who underwent abdominoplasty surgery can be kept alive in a special medium for 10 days, we focused on cell death changes in these samples.

**Figure 1:**
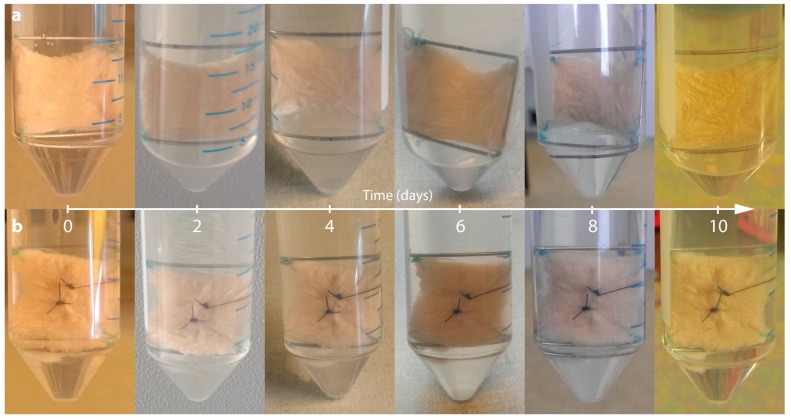
Photo coverage of full-thickness skin grafts of patient 1 (20 × 20 mm) fixed on frames made of surgical steel immediately after preparation (0 day). Pictures show samples immediately after explant dissection, as well as at 2, 4, 6, 8 and 10 days after mounting. All explants belong to the same donor. Non-wounded (**a**) and wounded simple interrupted sutured samples (**b**) are shown. No sample shows signs of necrosis, inflammation or disintegrated tissue.

The macroscopic overview of the skin samples with and without a suture revealed only some puckering and no visible signs of necrosis as demonstrated in Figure 1.

**Figure 2:**
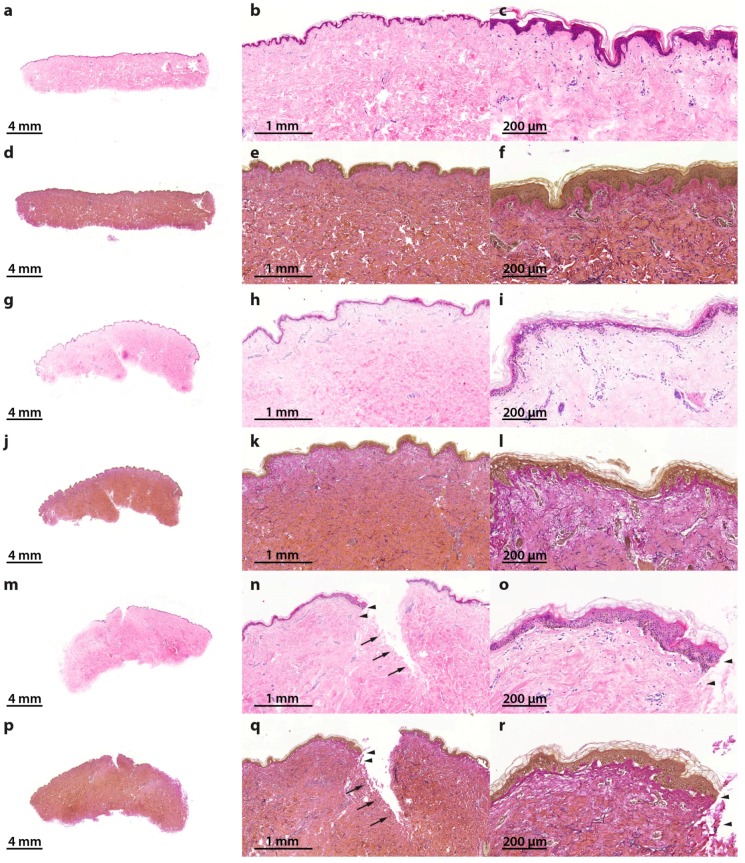
Hematoxylin–eosin (HE) and Elastica-van-Gieson (EvG) staining of patient 1 show vital skin structure at all times of investigation without necrosis or inflammation: reference samples, 0 day (HE (**a**–**c**); EvG (**d**–**f**); 0.5× (**a**,**d**), 3.5× (**b**,**e**) and 11.5× magnification (**c**,**f**)), 10 days non-wounded (HE (**g**–**i**); EvG (**j**–**l**); 0.5× (**g**,**j**), 3.5× (**h**,**k**) and 11.5× magnification (**i**,**l**)) and 10 days wounded (HE (**m**–**o**); EvG (**p**–**r**); 0.5× (**m**,**p**), 3.5× (**n**,**q**) and 11.5× magnification (**o**,**r**)). Wound edges are identified with arrows and arrowheads. Arrowheads show wound edge area at 11.5× magnification, in accordance with areas at 3.5× magnification.

Normally necrosis occurs when cells are exposed to a trauma followed by rapid disintegration. Apoptosis is a more orchestrated process that, in many cases, is functional to other biological processes. Necrosis and altered apoptosis mechanisms are detrimental and are indicative of a defective suture healing. Therefore, the presence of necrotic cells and apoptosis dysregulation will be analyzed in this suture model as a spaceflight preparatory study. For this purpose, the samples were embedded in paraffin and sections for histochemical staining were prepared. Hematoxylin–eosin (HE) staining [28,29] revealed no signs of necrosis in all specimens (Figure 2a–c,g–i,m–o). Elastica-van-Gieson staining [30] showed intact elastic fibers in the skin tissues cultured for 10 days in medium (Figure 2d–f,j–l,p–r).

Apoptosis is involved in the pathogenesis of a variety of diseases, such as cardiac injury [31], infections, transplant rejections, tumor responses to chemotherapy and/or radiotherapy and is also detectable in microgravity [31,32,33,34,35,36]. Nevertheless, apoptosis bears crucial functions in morphogenesis and organ development. The known mechanisms of apoptosis were reviewed by Schoenberger et al. [32] and Fuchs and Steller [37]. Here we use this knowledge to examine whether some of the inhibitors or inducers of programmed cell death are activated during the storage of the skin specimens and to investigate their gene expression pattern in ex vivo wound healing.

The morphological changes of programmed cell death were first described by Kerr et al. in 1972 [38]. The authors described alterations in cells different from necrotic alterations, such as cell shrinkage, chromatin condensation as well as membrane blebbing [38]. The process of apoptosis comprises a complex mechanism in which a large number of pro-apoptotic and anti-apoptotic factors interact in a specific manner. If present, necrotic areas together with inflammation and edema would have been detected easily by HE staining (Figure 2).

**Figure 3:**
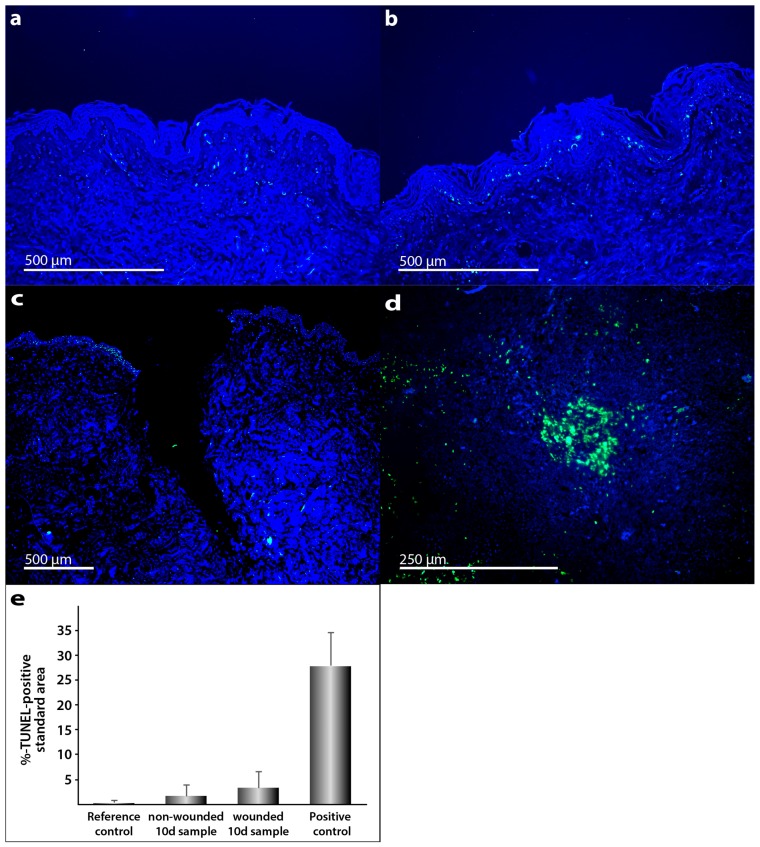
Detection of apoptosis in human full-thickness skin graft cross sections: TUNEL staining merged with DAPI counterstaining was used for apoptosis detection. Full-thickness skin grafts derived from explants from three patients who underwent abdominoplasty were investigated. Reference control (**a**) was taken at the day of surgery, 0 day. Apoptosis detection by fluorescence microscopy in unwounded (**b**) and wounded skin samples (**c**) after 10 days of organ culture. Palatine tonsil cross section was used as positive control (**d**). All shown skin samples are derived from patient 1. Semi-quantitative analysis of TUNEL-positive stained area (**e**). The standard deviation is indicated for each sample.

Morphological signs of apoptosis can be detected by application of the terminal deoxynucleotidyl transferase dUTP nick end labeling (TUNEL) technique. In addition, 4′,6-diamidino-2-phenylindole (DAPI) staining was performed (Figure 3a–d). We applied this method and demonstrated that only a few cells in the skin tissue samples became apoptotic during the culture. As positive control, human tonsil samples were used. These specimens normally reveal various apoptotic cells. Palatine tonsil positive controls (Figure 3d) show clear differences to TUNEL staining in the skin samples.

**Figure 4:**
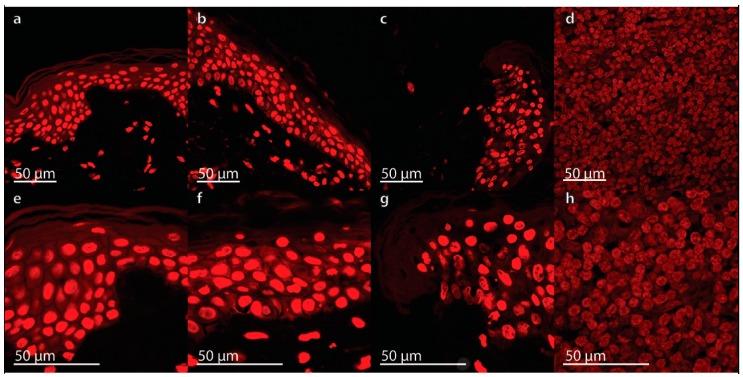
Propidium iodide (PI) staining in human full-thickness skin graft cross sections: No significant nuclear changes were detectable in the skin samples by PI-staining. A reference sample fixed at the day of surgery, 0 day (**a**,**e**), as well as a non-wounded (**b**,**f**) and wounded skin (**c**,**g**) sample cultured in organ culture medium for 10 days are shown. Palatine tonsil was used as positive control (**d**,**h**). All samples are shown in low (**a**–**d**) and high magnification (**e**–**h**).

In contrast, necrosis can be shown by propidium iodide (PI), a fluorescent intercalating agent used as a DNA stain. It is useful to detect necrotic cells [39]. Wounds caused by physical agents (trauma or burns) can become necrotic in case of bacterial infection or inflammation. Therefore, it is necessary to avoid infection and thus to prevent necrosis. Due to formaldehyde fixation of our cells, all cells are PI positive. The staining was utilized for morphological apoptosis assessment. We could show that no apoptosis related signs of morphological changes, like membrane blebbing, cell shrinkage or apoptotic bodies were visible in the skin samples. PI staining of the tissues is shown in Figure 4 and demonstrates no appearance of apoptosis in all specimens.

Taken together, the morphological investigations did not reveal any significant signs of necrosis. We could not detect any significant apoptosis in this experimental setting. To further examine the quality of the ex vivo skin samples, we focused on the gene expression level of apoptosis genes belonging to the extrinsic and intrinsic pathways of apoptosis.

### 2.2. Determination of the Gene Expression of Activators and Inhibitors of Apoptosis (Extrinsic and Intrinsic Pathways of Apoptosis) and Extracellular Matrix Components in Wound and Suture Models, Including Analysis of the Interaction Network of the Genes of Interest

#### 2.2.1. Gene Expression of Activators and Inhibitors of Apoptosis

Membrane-associated proteins like the death receptors FAS/CD95 and the receptors for the tumor necrosis factors (TNF-R) are involved in the extrinsic pathway of programmed cell death. They cause cell death after ligand-receptor binding [37,40,41,42]. The transcription of genes involved in the regulation of programmed cell death was investigated using quantitative real-time PCR (qPCR).

**Figure 5:**
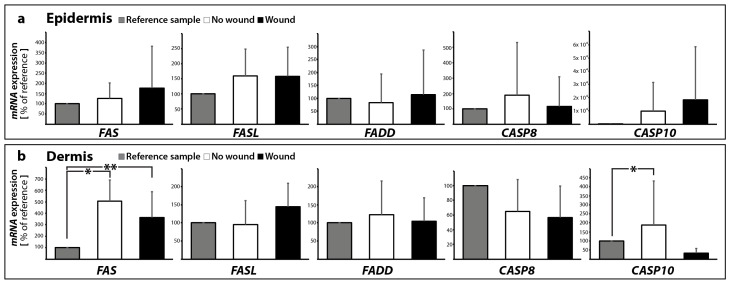
Quantitative real-time PCR of regulators of apoptosis: The mRNA expression levels are shown for reference control (0 day), non-wounded and wounded skin samples after ten days in organ culture medium. *FAS*, *FASL*, *FADD*, *CASP8*, and *CASP10* were investigated for epidermis (**a**) and dermis (**b**) respectively; * *p* < 0.05 reference vs. no wound, ** *p* < 0.05 reference vs. wound.

The investigation of the *FAS* (also called FAS-receptor) gene expression (Figure 5a,b) revealed no change in *FAS* mRNA in the wound sample (epidermis) compared to the control samples with no wound (0 and 10 days). In contrast, the *FAS* mRNA was significantly elevated in 10 days samples with and without a wound in the dermis compared with 0 day (Figure 5b). In addition, no significant changes were found for *FASL* in all groups in the epidermis and dermis (Figure 5a,b). FASL is the ligand for the FAS-receptor and its binding to FAS results in apoptosis mediated by caspase activation.

Interestingly, the *TNFA* and *TNFB* mRNAs were not detectable in all epidermis and dermis samples with/without wounds (data not shown). The binding of FASL results in FAS trimerization, which recruits the initiator caspase-8 via Fas-associated death domain protein (FADD). The *FADD* gene expression was not changed in all groups in the epidermis and dermis (Figure 5a,b).

A plasma membrane-associated death-inducing signaling complex (DISC) converts procaspase-8 into the mature active caspase-8 [43,44]. The activation of caspase-8 leads to the activation of the effector caspase-3 [44]. qPCR analyses revealed no significant changes in the *CASP8* mRNA expression between controls and sutured samples in the epidermis and dermis (Figure 5a,b).

Moreover, *CASP10* gene expression was not changed in the epidermis, whereas in the dermis a significant elevation of *CASP10* mRNA was measured in 10 days samples without a wound compared with 0 day (Figure 5a,b).

**Figure 6:**
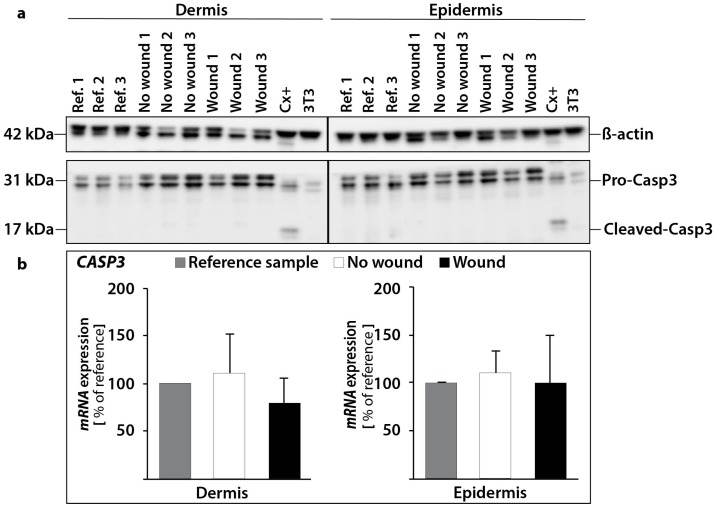
Investigation of caspase-3: changes in the protein content of pro-caspase-3 and cleaved caspase-3 (**a**). The amount of protein was investigated in reference controls (0 d), non-wounded and wounded samples. As a positive control for apoptosis CX+ cells were utilized. 3T3 protein was used as a cytoskeletal control. β-actin (loading control), pro-caspase-3 and cleaved caspase-3 were investigated independently in dermal and epidermal tissue for the same skin samples. Quantitative real-time PCR of *CASP3* gene expression revealed no changes (**b**).

The Western Blot analysis of caspase-3 revealed no activation of caspase-3 protein in the epidermis and dermis (Figure 6a). This result is consistent with our qPCR results for *CASP3*. We measured no significant changes for the *CASP3* gene expression in the epidermis and dermis. (Figure 6b).

Activated caspase-8 induces programmed cell death via two parallel cascades. The first option is the direct cleavage and activation of caspase-3. Secondly, it can cleave BID, a pro-apoptotic BCL2 family protein. The cleaved BID now termed truncated Bid (tBID) translocates to mitochondria, inducing cytochrome c release, which sequentially activates caspase-9 and -3.

**Figure 7:**
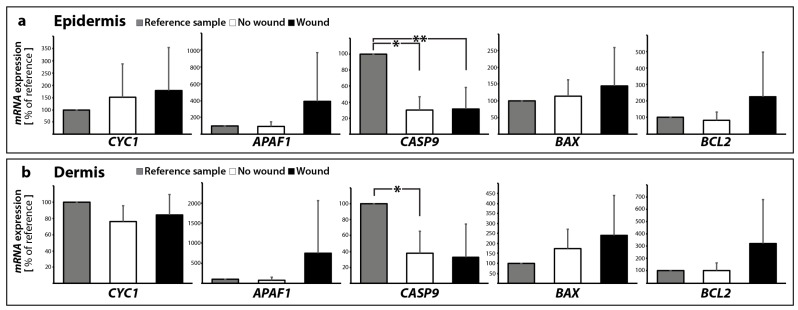
Quantitative real-time PCR of key factors involved in the intrinsic pathway of apoptosis: *CYC1*, *APAF1*, *CASP9*, *BAX*, and *BCL2* were investigated for epidermis (**a**) and dermis (**b**) samples, respectively; * *p* < 0.05 reference vs. no wound, ** *p* < 0.05 reference vs. wound.

Therefore, the next step was to focus on the gene expression of cytochrome c. These data are given in Figure 7a,b. The *CYC1* gene expression in the epidermis and dermis was not significantly altered. In addition, we examined the expression of the apoptotic protease activating factor 1, also known as *APAF1* (Figure 7a,b). It is involved in the cytochrome-c-dependent activation of caspase-3 (intrinsic pathway of apoptosis, as shown in Taylor et al. [45]). There was no significant change between no wound samples (0 and 10 days) in the epidermis and dermis. In the wound samples, there was a large standard deviation for *APAF1* detectable (Figure 7a,b).

Changes in the *CASP9* gene expression were generally characterized by down-regulation after 10 days compared with reference samples (Figure 7a,b).

BAX, the BCL2-associated X protein, stimulates the release of cytochrome c from mitochondria, while BCL2 is acting anti-apoptotic [46]. The *BAX* gene expression was not significantly changed in epidermis and dermis samples (Figure 7a,b). In addition, also the *BCL2* mRNA was not significantly changed in all samples of the 5 patients (Figure 7a,b).

**Figure 8:**
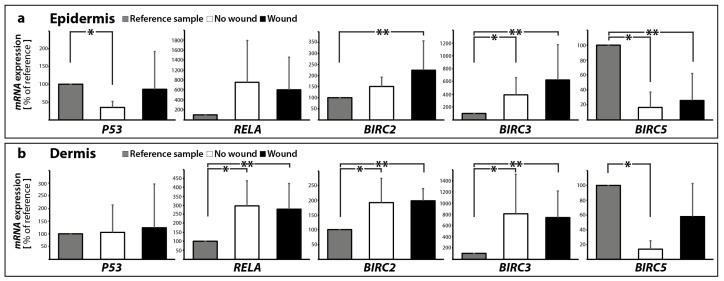
Quantitative real-time PCR of *P53*, *RELA*, *BIRC2*, *BIRC3* and *BIRC5* performed for epidermis (**a**) and dermis (**b**) samples; * *p* < 0.05 reference vs. no wound, ** *p* < 0.05 reference vs. wound.

We also investigated the tumor suppressor p53 (Figure 8a,b), which has various anticancer functions and is involved in apoptosis, genomic stability, and inhibition of angiogenesis [47]. It can activate DNA repair proteins, when the DNA has sustained damage. Thus, it may be also an important factor in aging. p53 can initiate programmed cell death if DNA damage proves to be irreparable [48]. Transient inhibition of p53 supports the early cell proliferation required for a rapid tissue repair [49].

In the epidermis, expression of *p53* was significantly downregulated in 10 days no wound samples compared to reference controls, 0 day (Figure 8a). In the dermis, there was no significant change measureable (Figure 8b).

In addition, we investigated the expression of NF-κB (nuclear factor κ-light-chain-enhancer of activated B cells), which is a protein complex that controls transcription of DNA, cytokine production and cell survival. NF-κB is involved in cellular responses to stimuli such as stress, cytokines, ultraviolet irradiation etc. NF-κB plays a key role in regulating the immune response to infection [50]. Incorrect regulation of NF-κB has been linked to cancer, inflammatory and autoimmune diseases, septic shock, viral infection, and improper immune development [51]. NF-κB incorporates a variety of transcriptional regulatory functions and is known to be of great importance in apoptosis. It is inactivated, by binding to I-κB (inhibitor of NF-κB). However, degradation of I-κB can result in a translocation of NF-κB into the nucleus, where it can activate the transcription of anti-apoptotic genes. The transcription factor p65, also known as the nuclear factor NF-κB p65 subunit, is a protein that in humans is encoded by the *RELA* gene. RELA is a REL-associated protein involved in NF-κB heterodimer formation, nuclear translocation and activation. NF-κB is an essential transcription factor complex involved in all types of cellular processes, including cellular metabolism, chemotaxis and more.

The data for *RELA* or NF-κB-subunit expression is given in Figure 8a,b. In the epidermis, *RELA* expression was slightly, but insignificantly elevated in 10 days samples with and without a wound (Figure 8a). In the dermis, *RELA* mRNA revealed a significant up-regulation in 10 days samples with and without a wound compared to 0 day (Figure 8b).

The presence of NF-κB subunits, such as *RELA*, in the epidermis inhibits epithelial cell death induced by Fas ligand, tumor necrosis factor-α, and microbes [52,53,54]. One mechanism by which NF-κB protects cells from apoptosis is the induction of expression of anti-apoptotic genes such as cellular inhibitors of apoptosis.

In a next step, we focused on an important part of the apoptotic machinery and investigated the inhibitor of apoptosis protein (IAP) family. IAPs are involved in regulating caspase activity, proliferation and survival of cells through binding to their baculovirus IAP repeat (BIR) domains [55]. First, we investigated the gene expression of *BIRC2*, which is the gene of the Baculoviral IAP repeat-containing protein 2. This multi-functional protein regulates not only caspases and apoptosis, but also modulates inflammatory signaling and immunity, mitogenic kinase signaling, and cell proliferation, as well as cell invasion and metastasis [56,57]. BIRC2 has been shown to interact with CASP9. Baculoviral IAP repeat-containing protein 2 inhibits apoptosis by binding to tumor necrosis factor receptor-associated factors TRAF1 and TRAF2, probably by interfering with activation of ICE-like proteases. BIRC 2 inhibits the activation of caspases-3, -7 and -9 [55].

We detected a significant increase in the *BIRC2* gene expression of wound tissues vs. 0 day controls in the epidermis (Figure 8a). In the dermis, the *BIRC2* mRNA was upregulated in both 10 dsya samples without and with wound compared with 0 day (Figure 8a).

Second, we also focused on *BIRC3.* The Baculoviral IAP repeat-containing protein 3 is a protein that in humans is encoded by the *BIRC3* gene. It is acting similar as BIRC2 and inhibits the activation of caspase-3 as well as -7 and -9 [55]. This gene encodes a member of the IAP family of proteins that inhibits apoptosis by binding to tumor necrosis factor receptor-associated factors TRAF1 and TRAF2. *BIRC3* gene expression patterns showed strong parallels to *BIRC2*. Interestingly, the samples showed significant increases in no wound and wound tissues vs. 0 day controls in both dermis and epidermis (Figure 8a,b).

Third, survivin, also called baculoviral inhibitor of apoptosis repeat-containing 5 or BIRC5, is a protein that, in humans, is encoded by the *BIRC5* gene. Survivin is a member of the inhibitor of apoptosis (IAP) family. The survivin protein inhibits caspase activation and functions as a negative regulator of programmed cell death. In addition, it also regulates the cell cycle [58]. Survivin acts on cytokinin and mitotic spindle formation to inhibit apoptosis [55]. The *BIRC5* gene is expressed in normal adult tissues, including skin, but also in cancer cells such as follicular thyroid cancer cells [35,59]. It is mostly detected in the nucleus of keratinocyte stem cells (KSCs) [60], but it is also expressed in melanocytes [61]. Survivin has an important role in the regulation of cell cycle [60], and is involved in promoting UV-induced melanoma development in vivo [61]. *BIRC5* was found to be downregulated, where significant changes in its gene expression were detected (Figure 8a) in epidermis samples. A significant decrease of *BIRC5* gene expression was found in no wound samples vs. 0 day controls in the dermis (Figure 8b).

**Figure 9:**
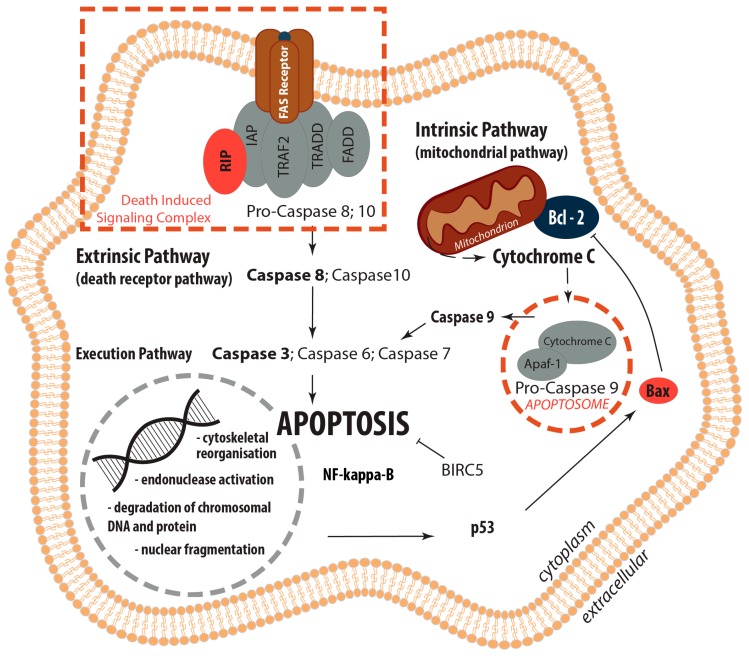
Overview: Regulation of Apoptosis. Extrinsic (death receptor pathway) and intrinsic (mitochondrial pathway) apoptosis pathways as well as their interplay within the common execution pathway are displayed.

These data clearly demonstrate no differential regulation of apoptosis genes of the extrinsic and intrinsic pathways of apoptosis in this ex vivo skin model after 10 days post-surgery (Figure 9). Histopathological investigations revealed no relevant damage of the skin samples.

#### 2.2.2. Gene Expression Profile of Extracellular Matrix Proteins

Laminins are glycoproteins and part of the collection of biochemical molecules that form the extracellular matrix (ECM). They are important for the control of biological processes, such as proliferation, differentiation, adhesion and migration [62]. They are key players in tissue homeostasis, wound healing and angiogenesis [63]. Laminins can be detected in all basal laminas. They possess binding sites to surface receptors. Together with collagen type IV, entactin and perlecan they form the basal lamina. Further players in the field are fibronectin and proteoglycans.

**Figure 10:**
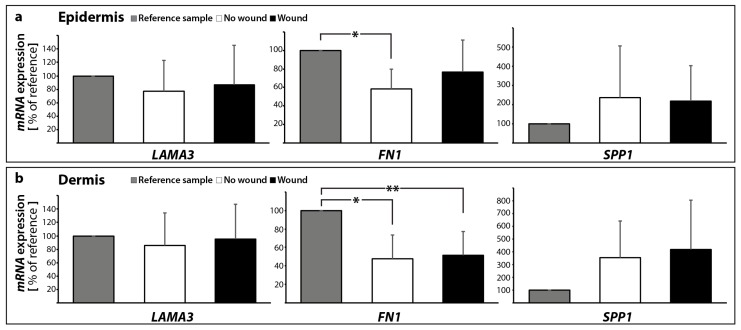
Gene expression patterns of selected extracellular matrix proteins: altered mRNA expression levels are shown for reference control (0 day), unwounded and wounded skin samples after ten days in organ culture medium. *LAMA3*, *FN1* and *SPP1* mRNAs were investigated for epidermis (**a**) and dermis (**b**) samples respectively; * *p* < 0.05 reference vs. no wound, ** *p* < 0.05 reference vs. wound.

Knowledge of the behavior of ECM proteins in wound healing processes is very important and has a great potential to help identifying new strategies for the enhancement of normal wound closure and the treatment of chronic/non-healing wounds. Here, we detected no significant change in the *LAMA3* gene expression in both epidermis and dermis samples with and without a suture after 10 days (Figure 10a,b).

The ECM protein fibronectin is involved in cell growth, migration, cell adhesion and differentiation processes. It plays an important role in wound healing and development. Changes in the expression of fibronectin have been reported in several diseases, such as diabetes, ischemia and reperfusion [64], as well as other cardiovascular disorders or cancers. Rats with diabetes revealed scarring and an increase in fibronectin-positive material in the cardiac left ventricular interstitium [28]. Moreover, it is known that intraluminal treatment with vascular endothelial growth factor (VEGF) is beneficial to the healing process in vascular microsurgery [65,66]. Fibronectin protein was elevated in a rat model with a vascular anastomosis and further induced by VEGF after 10 days post-surgery, which was paralleled by an elevation of osteopontin and various collagen subtypes [65]. The *FN1* mRNA was significantly decreased in the epidermis in the 10 days no wound sample, whereas no significant change was detectable to the wound (Figure 10a). The *FN1* mRNA was significantly reduced in both 10 days groups compared with the 0 day samples in the dermis (Figure 10b).

The expression of fibronectin and laminin, which are assessed in the phase of the experiment preparation to ascertain the viability of the tissue samples, will be analyzed in depth after the flight, because studies on fibroblasts and endothelial cells grown in simulated microgravity have shown marked alterations in production and three-dimensional organization of these ECM proteins, whose dysregulation could strongly affect the healing process [16].

Osteopontin (SPP1) belongs to the group of small integrin-binding ligand N-linked glycoproteins and acts as a cell adhesion molecule. Osteopontin is expressed in various cells such as bone cells, chondrocytes, endothelial cells and cancer cells. It is involved in several biological processes, where it mediates migration and cell adhesion. Moreover, osteopontin has also been connected to inflammation, where it is upregulated, atherosclerosis and the post-myocardial infarction remodeling process [67]. In our experimental setting, the *SPP1* gene expression was not changed in skin samples with and without a wound in the dermis and epidermis (Figure 10a,b).

This finding supports our further data and indicates that there is no apoptosis in the skin tissue cultured for 10 days ex vivo in medium. It has been reported that an elevated osteopontin expression is associated with increased myocyte apoptosis and myocardial dysfunction [68]. The authors showed that osteopontin is acting via CD44 receptors and induces apoptosis in myocytes via the involvement of the mitochondrial death pathway [68]. In summary, a concise overview of the qPCR results is given in Table 1.

#### 2.2.3. Interaction Network of the Genes of Interest

**Figure 11:**
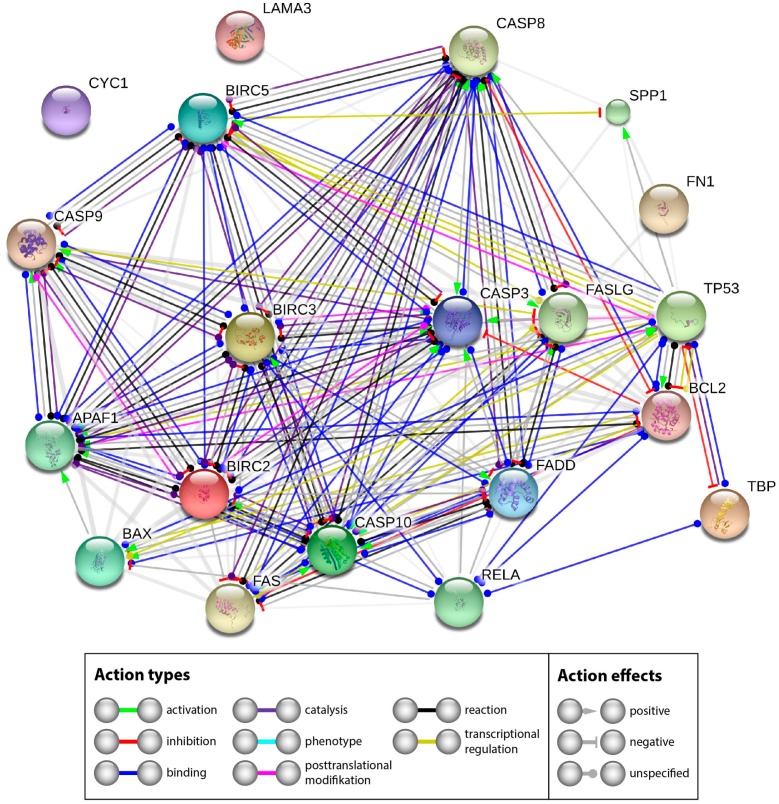
Mutual regulation of apoptosis related genes. STRING (Search Tool for the Retrieval of Interacting Genes/Proteins) network analysis shows functional interactions of genes and their products investigated.

The extensive investigation of apoptotic factors revealed no signs of an activation of intrinsic or extrinsic pathways of apoptosis—the *CASP3* expression was not differentially regulated. Western blot analyses revealed no cleaved caspase-3. A similar result was found for *CASP*9. The reason for the stability of Casp3 may be found by looking at the interaction network of the investigated genes and their products. Therefore, we performed a STRING network analysis to further investigate the functional interactions of genes and their products. The STRING analysis describes important features and the potential mechanisms behind ex vivo wound healing and possible apoptosis. Figure 11 shows that genes and their products, which are known to regulate apoptosis, interact on gene (yellow lines) and gene product levels in various ways of favoring and attenuating apoptosis. Their netting appears to be unaffected by *CYC1* and only weakly influenced by components of the extracellular matrix.

A strong regulatory effect is exerted by NF-κB. The *NF-κB* gene is upregulated and might have a pivotal role [69]. In our experiments, *RELA* seems to inhibit apoptosis via two routes. Its product binds to BCL2 (blue line), which is unregulated, but has a strong inhibitory effect on caspase-3 (red line with terminal cross bar). In addition, it binds BIRC2 and BIRC3, both are upregulated. BIRC 2 and 3 can inhibit caspase-3 (grey lines with terminal red cross bar). The other components of the network did not indicate significant regulation in our experiments. Hence it appears reasonable that the upregulated *RELA*, *BIRC2* and *BIRC3* genes could enforce the observed inhibition of apoptosis.

### 2.3. Design of the Analytical Protocols to Be Performed in the Future on Tissue Specimens after the Post-Spaceflight Download of the Samples

After the spaceflight, the samples will be transported to the laboratories of the science team by the ESA. There, photos from all samples will be taken to document the macroscopic view of the specimens and to check for damaged samples. Afterwards, histological investigations will be performed according to the procedures described in Section 2.1 to determine the morphological changes. In addition, the viability status of the skin samples cultured in space or on Earth will be checked (Section 2.1). The RNA*later*-fixed samples will be examined by quantitative qPCR to determine the gene expression of activators and inhibitors of apoptosis with a special focus on the intrinsic and extrinsic pathways of apoptosis (Section 2.2.1). In addition, the gene expression of ECM proteins will be measured (Section 2.2.2). Thereafter, the interaction of these genes of interest will be evaluated by STRING analyses (Section 2.2.3). These analytical protocols will be used post-spaceflight after download. 

## 3. Materials and Methods

### 3.1. Media, Supplements and Reagents

The following reagents were used to conduct the study: DMEM-F12 medium (Dulbecco’s modified eagle medium/F12 nutrient mixture (Ham), 1/1) with l-glutamine (Gibco by life technologies, Darmstadt, Germany); Dulbecco’s phosphate buffered saline (DPBS) with Ca^2+^ and Mg^2+^ (Gibco by life technologies). We supplemented the following additives to the medium: G-penicillin (Sigma-Aldrich, Munich, Germany); gentamicin (Sigma-Aldrich); amphotericin B (Gibco by life technologies); bovine serum albumin (Sigma-Aldrich); hydrocortisone (Sigma-Aldrich); insulin (Sigma-Aldrich) and L-ascorbic acid (Sigma-Aldrich). For transport or short storage, the tissue was stored in RPMI-1640 medium (Roswell Park Memorial Institute medium, Gibco by life technologies, Darmstadt, Germany) supplemented with lincomycin (lincomycin hydrochloride, Sigma Aldrich), colistin (colistin sulfate salt, Sigma-Aldrich) and vancomycin (Vancomycin HCL, AG Scientific. Inc., San Diego, CA, USA).

### 3.2. Tissue Preparation and Organ Culture

Full-thickness skin samples of five patients were obtained from abdominoplasty surgery. All patients gave their informed consent to include their explants into the study. The study was approved by the ethics committee of the Medical Faculty of the Otto-von-Guericke-University Magdeburg (Reference number AZ 128/16, 5 September 2016) and was conducted following rules of the local ethics committee. The tissue specimens included into this study were obtained from patients who underwent plastic surgery procedures after massive weight loss using gastric banding or comparable activities. The explant consisting of epidermis, dermis and subcutaneous tissue was stored in a sterile bag and maintained at 4 °C directly afterwards.

Immediately after the operation, the subcutaneous fat tissue was removed and previously dissected full-thickness skin grafts were rinsed in DPBS. For sample size standardization, the prepared samples were cut into squares with a side length of 20 mm. Thus, the surface area for each individual sample was about 400 mm^2^ (organ culture samples). Subsequently, all specimens were knotted on surgical steel frames to simulate physiological tissue tension (Ethicon (Norderstedt, Germany), Ethilon 4-0, Polyamide).

For tissue transportation, the entire explant was submerged in RPMI 1640 supplemented with 120 µg/mL lincomycin, 10 µg/mL colistin and 50 µg/mL vancomycin. On the first day of the study, a cross section was excised from each explant as a reference sample. Furthermore, epidermis and dermis tissues were prepared by surgical separation. Epidermis and dermis samples were snap-frozen in liquid nitrogen immediately after separation until further analysis. Cross sections were stored in 4% paraformaldehyde at room temperature until further histological assessment. For each patient half of all organ culture samples, already fixed on a frame, were wounded by sharp incision of 10 mm length and 2 mm depth. Subsequently, all lacerated skin samples were adapted with simple interrupted suture using 3-0 non-absorbable sutures (Ethicon, Ethilon 3-0, polyamide 6, black).

After mounting, lacerating and suturing the skin samples were placed in a culture chamber with culture medium composed of DMEM/Ham F12 supplemented with 8 μL/mL 20% bovine serum albumin, 0.4 μg/mL hydrocortisone, 0.12 IU/mL insulin, 100 IU/mL G penicillin, 20 μg/mL gentamycin, 1 μg/mL amphotericin B and 50 μg/mL ascorbic acid. The samples were stored at 32 °C. The organ culture medium was changed twice a week.

### 3.3. RNA Extraction and qPCR

Skin specimens of five patients were included in the study. The RNA from epidermis and dermis samples (*n* = 5 samples per group) was extracted using TRIzol^®^Reagent (Thermo Fischer Scientific). All samples were weighed. Subsequently, the extraction was performed as proposed by the manufacturer’s protocol [70]. The RNA content was checked for purity using a Nanodrop spectrophotometer (Thermo Scientific). cDNA synthesis and qPCR were performed as previously described by Kopp et al. [29]. qPCR was performed using the 7500 Real-Time PCR System (Applied Biosystems, Darmstadt, Germany). cDNA-selective-primers were synthesized by TIB Molbiol (Berlin, Germany) and are listed in Table 1. The primers (sequences given in Table 2) were designed using Primer Express (Applied Biosystems) to have a T_m_ of ~60 °C and to span exon-exon boundaries. All samples were measured in triplicate. For normalization, TATA-binding protein (TBP) was used as a housekeeping gene. The comparative *C*_T_ (∆∆*C*_T_) method was used for relative quantification of transcription levels and the 0 days group was defined as 100% for reference.

### 3.4. Histochemistry and Microscopy

Hematoxylin–eosin- and Elastica-van-Gieson-staining procedures were used to evaluate the tissue morphology for each cross section. The methods were published earlier [29]. All sections were visualized by light microscopy. The samples were investigated by microscopy using a LEICA DM2000 microscope equipped with a Leica DFC310 FX digital CCD color camera.

### 3.5. TUNEL Assay

TUNEL staining was done according to the manual provided by the manufacturer (Calbiochem^®^ (Merck Millipore, Darmstadt, Germany), FragEL™ DNA Fragmentation Detection Kit, Fluorescent—TdT Enzyme) [71]. Stained samples were investigated utilizing a Leica DM 2000 microscope connected to an external light source, Leica EL 6000 (Leica Microsystems GmbH, Wetzlar, Germany). Palatine tonsil cross-sections were taken as positive control using an objective with a calibrated magnification of 200×. Microscopic pictures of full-thickness skin grafts were created at 100× magnification.

### 3.6. Propidium Iodide Staining

For propidium iodide (PI) staining (Sigma-Aldrich) skin tissue specimens were fixed with paraformaldehyde and embedded in paraffin [72]. 5-µm sections were prepared. The slides were analyzed by confocal laser-scanning microscopy. Fluorescent staining was analyzed using a Zeiss LSM 710 CLSM (Zeiss, Jena, Germany) fitted with a Plan-Apochromat 63 × 1.4 objective as previously described [73]. The PI-stained slides were performed in triplicates.

### 3.7. Western Blot Analysis

Whole cell lysates were used for Western blotting following conventional protocols for gel electrophoresis and trans-blotting, as described earlier [31,65,66]. Equal amounts of 10 µL lysate containing 2 µg/µL protein were loaded on precast TGX stain-free gels (Bio-Rad, Munich, Germany). Transturbo blot PVDF membranes (Bio-Rad) were used for blotting. An overview of the used antibodies and their applied concentrations is given in Table 3. The analysis was performed using ImageQuant™ LAS 4000 (GE Healthcare UK Limited, Buckinghamshire, UK), and the densitometry was performed using ImageJ (NIH). In addition, we used protein from the colon carcinoma cell subline CX+ with high HSP70 as positive control for caspase-3 [74] and protein from NIH 3T3 fibroblasts, established from a NIH Swiss mouse embryo (Sigma-Aldrich).

### 3.8. STRING Analysis

Interactions between the components, whose genes were investigated, were determined using the STRING platform [75]. For each component, the UniProtKB entry number was inserted in the input form “multiple proteins” and “Homo sapiens” was selected as organism. The resulting network view was transferred to the interaction view [76,77] and downloaded. Yellow lines show transcriptional regulation, blue lines show binding, red lines indicate inhibition and black lines point to posttranslational modification.

### 3.9. Statistical Evaluation

Statistical Evaluation was performed using SPSS 24.0 (SPSS, Inc., Chicago, IL, USA). The Mann-Whitney-U-Test was used to compare the different conditions. All data is presented as mean ± standard deviation (SD) with a significance level of * *p* < 0.05. indicates the comparison of reference vs. non-wounded, ** indicates the comparison of reference vs. unwounded skin sample.

## 4. Conclusions

Regarding the first goal of the study, our data clearly demonstrate that it is possible to maintain live skin tissue cultures for 10 days on normal ground level and prove their applicability for a spaceflight experiment. This newly designed wound-healing model by our international space science team has proven to be suitable for future spaceflights. The viability of the tissue specimens was demonstrated, no significant signs of cell death (apoptosis/necrosis) were detected in no wound and sutured wound healing models. With respect to the gene expression analysis, comprising the second goal of the study, we comprehensively demonstrated the interplay of activators and inhibitors of apoptosis and extracellular components in ex vivo wound healing models. *FAS*, *CASP9* and *CASP10* (all apoptosis activators), *BIRC2*, *BIRC3* and *BIRC5* (apoptosis inhibitors), *RELA* as well as *FN1* (encoding an ECM protein) were significantly altered in the dermis of no wound samples. The expression of the genes *FN1*, *CASP9*, *P53*, *BIRC3* and *BIRC5* was also significantly altered in the epidermis of no wound samples. *CASP9*, *BIRC2*, *BIRC3* and *BIRC5* mRNAs were significantly changed in the epidermis of wound samples. In addition, *FN1*, *FAS*, *BIRC2*, *BIRC 3* and *RELA* mRNAs were significantly altered in the dermis of wound samples. Their interplay in the regulation of apoptosis and cell survival is given in Figure 9.

The STRING analysis demonstrated that NF-κB exerts a strong regulatory effect. As the *NF-κB* gene is upregulated, it might hence play an important role in the inhibition of apoptosis. Furthermore, the analysis suggested that the upregulated *RELA*, *BIRC2* and *BIRC3* genes could enforce the observed inhibition of apoptosis. Therefore, the data obtained from analyzing the key factors of the intrinsic and extrinsic pathways of apoptosis support the findings of goal 1. 

Concerning the third and last goal of our study, the performed methods in Section 2.1 and Section 2.2 demonstrated to be applicable for post-flight analyzes of tissue specimens after the download of the samples.

## Figures and Tables

**Table 1:** Fold changes in gene expression.

	Gene Symbol	No Wound		Wound	
		Epidermis	Dermis	Epidermis	Dermis
extrinsic pathway	*FASL*	1.59 ± 0.89	0.96 ± 0.65	1.58 ± 0.97	1.45 ± 0.65
	*FAS*	1.26 ± 0.76	5.08 ± 1.83 *	1.78 ± 2.04	3.63 ± 2.24 *
	*BIRC2*	1.51 ± 0.42	1.93 ± 0.82 *	2.25 ± 1.32 *	1.98 ± 0.42 *
	*BIRC3*	3.87 ± 2.70 *	8.10 ± 7.08 *	6.22 ± 5.50 *	7.41 ± 4.73 *
	*FADD*	0.82 ± 1.12	1.23 ± 0.93	1.14 ± 1.71	1.05 ± 0.65
	*CASP8*	1.88 ± 3.44	0.65 ± 0.43	1.18 ± 1.90	0.57 ± 0.43
	*CASP10*	48.7 ± 107.9	1.89 ± 2.43 *	90.8 ± 201.9	0.32 ± 0.26
	*CASP3*	1.10 ± 0.23	1.10 ± 0.42	1.00 ± 0.49	0.80 ± 0.26
intrinsic pathway	*BAX*	1.14 ± 0.49	1.73 ± 0.97	1.44 ± 1.16	2.41 ± 1.98
	*BCL2*	0.84 ± 0.48	1.02 ± 0.61	2.26 ± 2.70	3.23 ± 3.57
	*CYC1*	1.51 ± 1.37	0.76 ± 0.19	1.79 ± 1.77	0.84 ± 0.26
	*APAF1*	0.91 ± 0.57	0.81 ± 0.70	3.90 ± 5.81	7.53 ± 13.04
	*CASP9*	0.30 ± 0.17 *	0.38 ± 0.27 *	0.32 ± 0.27 *	0.33 ± 0.41
	*RELA*	7.53 ± 8.46	2.96 ± 1.40 *	6.06 ± 6.50	2.79 ± 1.43 *
	*BIRC5*	0.16 ± 0.21 *	0.14 ± 0.11 *	0.26 ± 0.36 *	0.58 ± 0.45
	*P53*	0.35 ± 0.17 *	1.06 ± 1.07	0.86 ± 1.06	1.24 ± 1.74
ECM	*LAMA3*	0.77 ± 0.45	0.86 ± 0.48	0.87 ± 0.58	0.95 ± 0.51
	*FN1*	0.59 ± 0.22 *	0.48 ± 0.25 *	0.77 ± 0.35	0.52 ± 0.26 *
	*SPP1*	2.35 ± 2.71	3.54 ± 2.85	2.18 ± 1.83	4.17 ± 3.88

* *p* < 0.05 vs. day 0 control. For no wound vs. wound no significant changes were found.

**Table 2:** Primer sequences for qPCR.

Gene	Primer Name	Sequence
*TBP*	TATA-F	GTGACCCAGCATCACTGTTTC
	TATA-R	GCAAACCAGAAACCCTTGCG
*APAF1*	APAF1-F	GGAGGATATATTAAGTGGTGGAACG
	APAF1-R	GTTTTGAAGTCAGGGGACACG
*BAX*	BAX-F	GTCAGCTGCCACTCGGAAA
	BAX-R	AGTAACATGGAGCTGCAGAGGAT
*BCL2*	BCL2-F	CCTGTGGATGACTGAGTACCTGAA
	BCL2-R	TCAGAGACAGCCAGGAGAAATCA
*BIRC2*	BIRC2-F	GCTTTTGTTGTGATGGTGGCT
	BIRC2-R	ACTCACACCTTGGAAACCACT
*BIRC3*	BIRC3-F	TGCTGTGATGGTGGACTCAG
	BIRC3-R	ACTCACACCTTGGAAACCACT
*BIRC5*	BIRC5-F	GCCAGATGACGACCCCATAG
	BIRC5-R	CACCAAGGGTTAATTCTTCAAACTG
*CASP3*	CASP3-F	AACTGCTCCTTTTGCTGTGATCT
	CASP3-R	GCAGCAAACCTCAGGGAAAC
*CASP8*	CASP8-F	TGCAAAAGCACGGGAGAAAG
	CASP8-R	CTCTTCAAAGGTCGTGGTCAAAG
*CASP9*	CASP9-F	CTCCAACATCGACTGTGAGAAGTT
	CASP9-R	GCGCCAGCTCCAGCAA
*CASP10*	CASP10-F	GACAAGGAAGCCGAGTCGTA
	CASP10-R	GCCTCTGTGGTTCCGATTCA
*CYC1*	CYC1-F	CACTGCGGGAAGGTCTCTAC
	CYC1-R	GGGGTGCCATCGTCAAACTC
*FADD*	FADD-F	CCTGGGGAAGAAGACCTGTGTG
	FADD-R	TCGATGCTGTCGATCTTGGTG
*FAS*	FAS-F	AGTCTGGTTCATCCCCATTGAC
	FAS-R	AGGGATTGGAATTGAGGAAGACT
*FASL*	FASL-F	CTTCACTCCAGAAAGCAGGACAAT
	FASL-R	AGGGATTGGAATTGAGGAAGACT
*FN1*	FN1-F	AGATCTACCTGTACACCTTGAATGACA
	FN1-R	CATGATACCAGCAAGGAATTGG
*LAMA3*	LAMA3-F	AAAGCAAGAAGTCAGTCCAGC
	LAMA3-R	TCCCATGAAGACCATCTCGG
*P53*	P53-F	CCTGGATTGGCCAGACTGC
	P53-R	TTTTCAGGAAGTAGTTTCCATAGGT
*RELA*	RELA-F	CGCTTCTTCACACACTGGATTC
	RELA-R	ACTGCCGGGATGGCTTCT
*SPP1*	SPP1-F	CGAGGTGATAGTGTGGTTTATGGA
	SPP1-R	CGTCTGTAGCATCAGGGTACTG

All sequences are given in 5′-3′ direction.

**Table 3:** Antibodies applied for Western blot analysis.

Primary Antibody	Company	Dilution	Molecular Weight (kDa)	Catalogue Number
Caspase-3	Abcam	1:1000	31, 21–14	Ab13585
β-actin	Sigma	1:1000	42	A5316

The corresponding secondary antibodies—HRP-linked anti-mouse IgG (#7076) and anti-rabbit IgG (#7074, both Cell Signaling Technology, Danvers, MA, USA)—were used at a dilution of 1:3000.

## Abbreviations

APAF-1	Apoptotic protease-activating factor 1
BIRC5	Apoptosis inhibitor surviving
*BAX*	Apoptosis regulator Bax (BCL2 associated athanogene) gene
*BCL2*	Apoptosis regulator Bcl-2 (B-cell lymphoma protein 2) gene
Caspase-3	Cysteinyl aspartic acid-protease-3
Caspase-6	Cysteinyl aspartic acid-protease-6
Caspase-7	Cysteinyl aspartic acid-protease-7
Caspase-8	Cysteinyl aspartic acid-protease-8
Caspase-9	Cysteinyl aspartic acid-protease-9
Caspase-10	Cysteinyl aspartic acid-protease-10
*CYC1*	Cytochrome C gene
DAPI	4′,6-Diamidin-2-phenylindol
ECM	Extracellular Matrix
FADD	Fas-associated death domain
FAS/CD95	Tumor necrosis factor receptor superfamily member 6
*FN1*	Fibronectin gene
IAP	Inhibitor of Apoptosis Proteins
RIP	Cell death protein RIP
TNFA	Tumor necrosis factor α
TNFB	Lymphotoxin-α
TRAF2	TNF receptor-associated factor 2
TRAIL	Tumor necrosis factor ligand superfamily member 10
TUNEL	Terminal deoxynucleotidyl transferase dUTP nick end labeling

## References

[1] Singer A.J., Clark R.A. (1999). Cutaneous wound healing. N. Engl. J. Med..

[2] Eming S.A., Martin P., Tomic-Canic M. (2014). Wound repair and regeneration: Mechanisms, signaling, and translation. Sci. Transl. Med..

[3] Pasparakis M., Haase I., Nestle F.O. (2014). Mechanisms regulating skin immunity and inflammation. Nat. Rev. Immunol..

[4] Reinke J.M., Sorg H. (2012). Wound repair and regeneration. Eur. Surg. Res..

[5] Darby I.A., Laverdet B., Bonte F., Desmouliere A. (2014). Fibroblasts and myofibroblasts in wound healing. Clin. Cosmet. Investig. Dermatol..

[6] Greenhalgh D.G. (1998). The role of apoptosis in wound healing. Int. J. Biochem. Cell Biol..

[7] Huang N.F., Zac-Varghese S., Luke S. (2003). Apoptosis in skin wound healing. Wounds.

[8] Drudi L., Ball C.G., Kirkpatrick A.W., Saary J., Grenon S.M. (2012). Surgery in space: Where are we at now?. Acta Astronaut..

[9] Davidson J.M., Aquino A.M., Woodward S.C., Wilfinger W.W. (1999). Sustained microgravity reduces intrinsic wound healing and growth factor responses in the rat. FASEB J..

[10] Campbell M.R., Williams D.R., Buckey J.C., Kirkpatrick A.W. (2005). Animal surgery during spaceflight on the Neurolab Shuttle mission. Aviat. Space Environ. Med..

[11] Midura R.J., Su X., Androjna C. (2006). A simulated weightlessness state diminishes cortical bone healing responses. J. Musculoskelet. Neuronal Interact..

[12] Delp M.D. (2008). Unraveling the complex web of impaired wound healing with mechanical unloading and physical deconditioning. J. Appl. Physiol..

[13] Radek K.A., Baer L.A., Eckhardt J., DiPietro L.A., Wade C.E. (2008). Mechanical unloading impairs keratinocyte migration and angiogenesis during cutaneous wound healing. J. Appl. Physiol..

[14] Heinemeier K.M., Olesen J.L., Haddad F., Schjerling P., Baldwin K.M., Kjaer M. (2009). Effect of unloading followed by reloading on expression of collagen and related growth factors in rat tendon and muscle. J. Appl. Physiol..

[15] Morbidelli L., Monici M., Marziliano N., Cogoli A., Fusi F., Waltenberger J., Ziche M. (2005). Simulated hypogravity impairs the angiogenic response of endothelium by up-regulating apoptotic signals. Biochem. Biophys. Res. Commun..

[16] Monici M., Cialdai F., Romano G., Fusi F., Egli M., Pezzatini S., Morbidelli L. (2011). An in Vitro Study on Tissue Repair: Impact of Unloading on Cells Involved in the Remodelling Phase. Microgravity Sci. Technol..

[17] Cialdai F., Vignali L., Morbidelli L., Colciago A., Celotti F., Santi A., Caselli A., Cirri P., Monici M. (2017). Modeled Microgravity Affects Fibroblast Functions Related to Wound Healing. Microgravity Sci. Technol..

[18] Hautier A., Sabatier F., Stellmann P., Andrac L., De Gorce Y.N., Dignat-George F., Magalon G. (2008). Assessment of organ culture for the conservation of human skin allografts. Cell Tissue Bank..

[19] Gawlitta D., Oomens C.W., Baaijens F.P., Bouten C.V. (2004). Evaluation of a continuous quantification method of apoptosis and necrosis in tissue cultures. Cytotechnology.

[20] Elson K.M., Fox N., Tipper J.L., Kirkham J., Hall R.M., Fisher J., Ingham E. (2015). Non-destructive monitoring of viability in an ex vivo organ culture model of osteochondral tissue. Eur. Cell Mater..

[21] Lenselink E.A. (2015). Role of fibronectin in normal wound healing. Int. Wound J..

[22] Rai N.K., Tripathi K., Sharma D., Shukla V.K. (2005). Apoptosis: A basic physiologic process in wound healing. Int. J. Low. Extrem. Wounds.

[23] Wu Y.S., Chen S.N. (2014). Apoptotic cell: Linkage of inflammation and wound healing. Front. Pharmacol..

[24] Kane C.D., Greenhalgh D.G. (2000). Expression and localization of p53 and BCL-2 in healing wounds in diabetic and nondiabetic mice. Wound Repair Regen..

[25] Kleszczynski K., Fischer T.W. (2012). Development of a short-term human full-thickness skin organ culture model in vitro under serum-free conditions. Arch. Dermatol. Res..

[26] Oliver N., Babu M., Diegelmann R. (1992). Fibronectin gene transcription is enhanced in abnormal wound healing. J. Investig. Dermatol..

[27] Singh P., Carraher C., Schwarzbauer J.E. (2010). Assembly of fibronectin extracellular matrix. Annu. Rev. Cell Dev. Biol..

[28] Grimm D., Jabusch H.C., Kossmehl P., Huber M., Fredersdorf S., Griese D.P., Kramer B.K., Kromer E.P. (2002). Experimental diabetes and left ventricular hypertrophy: Effects of beta-receptor blockade. Cardiovasc. Pathol..

[29] Kopp S., Slumstrup L., Corydon T.J., Sahana J., Aleshcheva G., Islam T., Magnusson N.E., Wehland M., Bauer J., Infanger M. (2016). Identifications of novel mechanisms in breast cancer cells involving duct-like multicellular spheroid formation after exposure to the Random Positioning Machine. Sci. Rep..

[30] Grimm D., Infanger M., Westphal K., Ulbrich C., Pietsch J., Kossmehl P., Vadrucci S., Baatout S., Flick B., Paul M. (2009). Adelayed type of three-dimensional growth of human endothelial cells under simulated weightlessness. Tissue Eng. Part A.

[31] Kossmehl P., Kurth E., Faramarzi S., Habighorst B., Shakibaei M., Wehland M., Kreutz R., Infanger M., AH J.D., Grosse J. (2006). Mechanisms of apoptosis after ischemia and reperfusion: Role of the renin-angiotensin system. Apoptosis.

[32] Schoenberger J., Bauer J., Moosbauer J., Eilles C., Grimm D. (2008). Innovative strategies in in vivo apoptosis imaging. Curr. Med. Chem..

[33] Pohl F., Grosse J., Grimm D., Brockhoff G., Westphal K., Moosbauer J., Koelbl O., Infanger M., Eilles C., Schoenberger J. (2010). Changes of apoptosis, p53, and BCL-2 by irradiation in poorly differentiated thyroid carcinoma cell lines: A prognostic marker for the prospect of therapeutic success?. Thyroid.

[34] Wehland M., Ma X., Braun M., Hauslage J., Hemmersbach R., Bauer J., Grosse J., Infanger M., Grimm D. (2013). The impact of altered gravity and vibration on endothelial cells during a parabolic flight. Cell. Physiol. Biochem..

[35] Grosse J., Warnke E., Wehland M., Pietsch J., Pohl F., Wise P., Magnusson N.E., Eilles C., Grimm D. (2014). Mechanisms of apoptosis in irradiated and sunitinib-treated follicular thyroid cancer cells. Apoptosis.

[36] Ma X., Pietsch J., Wehland M., Schulz H., Saar K., Hubner N., Bauer J., Braun M., Schwarzwalder A., Segerer J. (2014). Differential gene expression profile and altered cytokine secretion of thyroid cancer cells in space. FASEB J..

[37] Fuchs Y., Steller H. (2015). Live to die another way: Modes of programmed cell death and the signals emanating from dying cells. Nat. Rev. Mol. Cell Biol..

[38] Kerr J.F., Wyllie A.H., Currie A.R. (1972). Apoptosis: A basic biological phenomenon with wide-ranging implications in tissue kinetics. Br. J. Cancer.

[39] Cummings B.S., Schnellmann R.G. (2004). Measurement of cell death in mammalian cells. Curr. Protoc. Pharmacol..

[40] Ashkenazi A., Dixit V.M. (1998). Death receptors: Signaling and modulation. Science.

[41] Itoh N., Yonehara S., Ishii A., Yonehara M., Mizushima S., Sameshima M., Hase A., Seto Y., Nagata S. (1991). The polypeptide encoded by the cDNA for human cell surface antigen Fas can mediate apoptosis. Cell.

[42] Yonehara S., Ishii A., Yonehara M. (1989). Acell-killing monoclonal antibody (anti-Fas) to a cell surface antigen co-downregulated with the receptor of tumor necrosis factor. J. Exp. Med..

[43] Yang J.K. (2015). Death effecter domain for the assembly of death-inducing signaling complex. Apoptosis.

[44] Siegmund D., Lang I., Wajant H. (2017). Cell death-independent activities of the death receptors CD95, TRAILR1, and TRAILR2. FEBS J..

[45] Taylor R.C., Cullen S.P., Martin S.J. (2008). Apoptosis: Controlled demolition at the cellular level. Nat. Rev. Mol. Cell Biol..

[46] Tsujimoto Y. (1998). Role of BCL-2 family proteins in apoptosis: Apoptosomes or mitochondria?. Genes Cells.

[47] Haupt S., Berger M., Goldberg Z., Haupt Y. (2003). Apoptosis—The p53 network. J. Cell Sci..

[48] Giaccia A.J., Kastan M.B. (1998). The complexity of p53 modulation: Emerging patterns from divergent signals. Genes Dev..

[49] Vollmar B., El-Gibaly A.M., Scheuer C., Strik M.W., Bruch H.P., Menger M.D. (2002). Acceleration of cutaneous wound healing by transient p53 inhibition. Lab. Investig..

[50] Hayden M.S., West A.P., Ghosh S. (2006). NF-κB and the immune response. Oncogene.

[51] Siebenlist U., Franzoso G., Brown K. (1994). Structure, regulation and function of NF-κB. Annu. Rev. Cell Biol..

[52] Qin J.Z., Chaturvedi V., Denning M.F., Choubey D., Diaz M.O., Nickoloff B.J. (1999). Role of NF-κB in the apoptotic-resistant phenotype of keratinocytes. J. Biol. Chem..

[53] Seitz C.S., Freiberg R.A., Hinata K., Khavari P.A. (2000). NF-κB determines localization and features of cell death in epidermis. J. Clin. Investig..

[54] Li M., Shillinglaw W., Henzel W.J., Beg A.A. (2001). The Rela(p65) subunit of NF-κB is essential for inhibiting double-stranded RNA-induced cytotoxicity. J. Biol. Chem..

[55] Saleem M., Qadir M.I., Perveen N., Ahmad B., Saleem U., Irshad T., Ahmad B. (2013). Inhibitors of apoptotic proteins: New targets for anticancer therapy. Chem. Biol. Drug Des..

[56] Dubrez-Daloz L., Dupoux A., Cartier J. (2008). IAPs: More than just inhibitors of apoptosis proteins. Cell Cycle.

[57] Yang Y.L., Li X.M. (2000). The IAP family: Endogenous caspase inhibitors with multiple biological activities. Cell Res..

[58] Zangemeister-Wittke U., Simon H.U. (2004). An IAP in action: The multiple roles of survivin in differentiation, immunity and malignancy. Cell Cycle.

[59] Dallaglio K., Marconi A., Pincelli C. (2012). Survivin: A dual player in healthy and diseased skin. J. Investig. Dermatol..

[60] Marconi A., Dallaglio K., Lotti R., Vaschieri C., Truzzi F., Fantini F., Pincelli C. (2007). Survivin identifies keratinocyte stem cells and is downregulated by anti-beta1 integrin during anoikis. Stem Cells.

[61] Thomas J., Liu T., Cotter M.A., Florell S.R., Robinette K., Hanks A.N., Grossman D. (2007). Melanocyte expression of survivin promotes development and metastasis of UV-induced melanoma in HGF-transgenic mice. Cancer Res..

[62] Ulbrich C., Pietsch J., Grosse J., Wehland M., Schulz H., Saar K., Hubner N., Hauslage J., Hemmersbach R., Braun M. (2011). Differential gene regulation under altered gravity conditions in follicular thyroid cancer cells: Relationship between the extracellular matrix and the cytoskeleton. Cell. Physiol. Biochem..

[63] Iorio V., Troughton L.D., Hamill K.J. (2015). Laminins: Roles and Utility in Wound Repair. Adv. Wound Care.

[64] Kossmehl P., Schonberger J., Shakibaei M., Faramarzi S., Kurth E., Habighorst B., von Bauer R., Wehland M., Kreutz R., Infanger M. (2005). Increase of fibronectin and osteopontin in porcine hearts following ischemia and reperfusion. J. Mol. Med..

[65] Infanger M., Shakibaei M., Kossmehl P., Hollenberg S.M., Grosse J., Faramarzi S., Schulze-Tanzil G., Paul M., Grimm D. (2005). Intraluminal application of vascular endothelial growth factor enhances healing of microvascular anastomosis in a rat model. J. Vasc. Res..

[66] Infanger M., Grosse J., Westphal K., Leder A., Ulbrich C., Paul M., Grimm D. (2008). Vascular endothelial growth factor induces extracellular matrix proteins and osteopontin in the umbilical artery. Ann. Vasc. Surg..

[67] Lund S.A., Giachelli C.M., Scatena M. (2009). The role of osteopontin in inflammatory processes. J. Cell Commun. Signal..

[68] Dalal S., Zha Q., Daniels C.R., Steagall R.J., Joyner W.L., Gadeau A.P., Singh M., Singh K. (2014). Osteopontin stimulates apoptosis in adult cardiac myocytes via the involvement of CD44 receptors, mitochondrial death pathway, and endoplasmic reticulum stress. Am. J. Physiol. Heart Circ. Physiol..

[69] Grosse J., Wehland M., Pietsch J., Schulz H., Saar K., Hubner N., Eilles C., Bauer J., Abou-El-Ardat K., Baatout S. (2012). Gravity-sensitive signaling drives 3-dimensional formation of multicellular thyroid cancer spheroids. FASEB J..

[70] Chomczynski P. (1993). Areagent for the single-step simultaneous isolation of RNA, DNA and proteins from cell and tissue samples. BioTechniques.

[71] Darzynkiewicz Z., Juan G., Li X., Gorczyca W., Murakami T., Traganos F. (1997). Cytometry in cell necrobiology: Analysis of apoptosis and accidental cell death (necrosis). Cytometry.

[72] Simon D., Hoesli S., Roth N., Staedler S., Yousefi S., Simon H.U. (2011). Eosinophil extracellular DNA traps in skin diseases. J. Allergy Clin. Immunol..

[73] Dollerup P., Thomsen T.M., Nejsum L.N., Faerch M., Osterbrand M., Gregersen N., Rittig S., Christensen J.H., Corydon T.J. (2015). Partial nephrogenic diabetes insipidus caused by a novel AQP2 variation impairing trafficking of the aquaporin-2 water channel. BMC Nephrol..

[74] Gehrmann M., Marienhagen J., Eichholtz-Wirth H., Fritz E., Ellwart J., Jaattela M., Zilch T., Multhoff G. (2005). Dual function of membrane-bound heat shock protein 70 (Hsp70), Bag-4, and Hsp40: Protection against radiation-induced effects and target structure for natural killer cells. Cell Death Differ..

[75] Snel B., Lehmann G., Bork P., Huynen M.A. (2000). STRING: A web-server to retrieve and display the repeatedly occurring neighbourhood of a gene. Nucleic Acids Res..

[76] Riwaldt S., Pietsch J., Sickmann A., Bauer J., Braun M., Segerer J., Schwarzwalder A., Aleshcheva G., Corydon T.J., Infanger M. (2015). Identification of proteins involved in inhibition of spheroid formation under microgravity. Proteomics.

[77] Pietsch J., Riwaldt S., Bauer J., Sickmann A., Weber G., Grosse J., Infanger M., Eilles C., Grimm D. (2013). Interaction of proteins identified in human thyroid cells. Int. J. Mol. Sci..

